# Development and initial evaluation of a treatment decision dashboard

**DOI:** 10.1186/1472-6947-13-51

**Published:** 2013-04-21

**Authors:** James G Dolan, Peter J Veazie, Ann J Russ

**Affiliations:** 1 Department of Public Health Sciences, University of Rochester School of Medicine & Dentistry, 265 Crittenden Blvd. CU420644, Rochester, NY 14642, USA; 2 Department of Psychiatry, University of Rochester School of Medicine & Dentistry, 300 E. River Road, Box 278703, Rochester, NY 14627, USA

## Abstract

**Background:**

For many healthcare decisions, multiple alternatives are available with different combinations of advantages and disadvantages across several important dimensions. The complexity of current healthcare decisions thus presents a significant barrier to informed decision making, a key element of patient-centered care.

Interactive decision dashboards were developed to facilitate decision making in Management, a field marked by similarly complicated choices. These dashboards utilize data visualization techniques to reduce the cognitive effort needed to evaluate decision alternatives and a non-linear flow of information that enables users to review information in a self-directed fashion. Theoretically, both of these features should facilitate informed decision making by increasing user engagement with and understanding of the decision at hand. We sought to determine if the interactive decision dashboard format can be successfully adapted to create a clinically realistic prototype patient decision aid suitable for further evaluation and refinement.

**Methods:**

We created a computerized, interactive clinical decision dashboard and performed a pilot test of its clinical feasibility and acceptability using a multi-method analysis. The dashboard summarized information about the effectiveness, risks of side effects and drug-drug interactions, out-of-pocket costs, and ease of use of nine analgesic treatment options for knee osteoarthritis. Outcome evaluations included observations of how study participants utilized the dashboard, questionnaires to assess usability, acceptability, and decisional conflict, and an open-ended qualitative analysis.

**Results:**

The study sample consisted of 25 volunteers - 7 men and 18 women - with an average age of 51 years. The mean time spent interacting with the dashboard was 4.6 minutes. Mean evaluation scores on scales ranging from 1 (low) to 7 (high) were: mechanical ease of use 6.1, cognitive ease of use 6.2, emotional difficulty 2.7, decision-aiding effectiveness 5.9, clarification of values 6.5, reduction in decisional uncertainty 6.1, and provision of decision-related information 6.0. Qualitative findings were similarly positive.

**Conclusions:**

Interactive decision dashboards can be adapted for clinical use and have the potential to foster informed decision making. Additional research is warranted to more rigorously test the effectiveness and efficiency of patient decision dashboards for supporting informed decision making and other aspects of patient-centered care, including shared decision making.

## Background

An important component of quality healthcare is patient-centeredness, defined by the Institute of Medicine as “… care that is respectful of and responsive to individual patient preferences, needs, and values and ensuring that patient values guide all clinical decisions” [1]. Despite its importance, providing patient-centered care is a complex task that involves integrating clinical evidence, biomedical data, and other technical information with patients’ personal preferences, circumstances, and values.

Patient decision aids are intended to facilitate this process. They are “evidence-based tools designed to prepare clients to participate in making specific and deliberated choices among healthcare options” [2]. Patient decision aids have been shown to increase patients’ knowledge, reduce decisional conflict, and foster patient involvement in decisions about their care [2-4]. However, despite these promising results and growing interest in their use, the most effective way(s) to design and build patient decision aids is unknown, their effect on outcomes is uncertain, and we do not know how to most effectively integrate patient decision aids into busy practice settings [5-7].

A key challenge for patient decision aids is to help patients make sense of new, unfamiliar information about alternative management strategies so that they can make informed judgments about which ones they prefer [8]. Increasingly, healthcare decisions involve multiple alternatives with varying combinations of advantages and disadvantages. The presentation of unfamiliar information can lead to an incomplete understanding of the decision task and the consideration of multiple alternatives across various attributes is a substantial cognitive load for decision makers. Circumstances like these can cause people to make sub-optimal decisions or even avoid making a choice [9,10].

The need to quickly and effectively integrate large amounts of information across several dimensions is not unique to health care decisions. Busy decision makers in many different contexts face similar challenges. Advances in cognitive science and computer technology have led to increasing interest in the use of interactive visual information displays to support decision making in these circumstances [11,12]. One of the first products of these activities is the computer-based, interactive decision dashboard. Interactive decision dashboards are being increasingly used in business settings [13]. In healthcare, dashboards have been used to advance quality improvement [14-17], medication safety [18], intensive care unit patient management [19], implementation and monitoring of mental health care guidelines [20], and patient wellness [21].

A decision dashboard is “… a visual display of the most important information needed to achieve one or more objectives; consolidated and arranged on a single screen so the information can be monitored at a glance” [13]. The key components of decision dashboards include: a visual summary of decision-related information displayed in a single view, extensive use of graphical information displays, and features that allow users to easily interact with and explore the information being presented. These components allow dashboards to quickly communicate information about the pros and cons of decision alternatives by reducing the cognitive effort required by structuring the decision, highlighting factors that merit consideration, making the information more evaluable by actively engaging well-developed human visual capabilities to help people process and understand information, and providing information in a non-linear format to facilitate its incorporation in decision making deliberations [12,13,22-29]. Additionally, because dashboards allow users to control the extent and content of information displayed, they provide a means for users to self-regulate information exposure and avoid overload in the face of large amounts of information [22]. Theoretically, clinical decision dashboards that are properly formatted to take maximum advantage of inherent human visual and cognitive capabilities could provide a way to promote informed decision making effectively and efficiently [11].

The similarity between the purpose of a patient decision aid and the functionality of interactive decision dashboards suggests the dashboard format could be a useful way to create decision aids capable of facilitating informed decision making about complex, unfamiliar healthcare issues and promoting patient-centered care in busy clinical settings. To our knowledge, however, the use of interactive dashboards to support clinical decision making has not been explored. The goal of this study was determine if the interactive decision dashboard format can be successfully adapted to create a clinically realistic and feasible patient decision aid prototype suitable for further refinement and evaluation.

## Methods

Patient decision aids are complex health interventions. The objective of this initial study was to complete phases one and two - theoretical development and development of the intervention - of the five phase methodology for studying the effects of complex health interventions proposed by the British Medical Research Council [30,31].

### Development of the prototype clinical decision dashboard

We based our clinical dashboard prototype on a patient decision aid regarding selection of non-opioid pain medication for treatment of osteoarthritis pain produced by the Agency for HealthCare Research and Quality (AHRQ) [32,33]. We chose this example because decisions regarding medication use, particularly for patients with chronic illnesses, are among the most common clinical decisions made and frequently depend on individual patient preferences and circumstances.

The AHRQ brochure contains information about the beneficial effects of non-opioid analgesics on joint pain and swelling, risks of stomach bleeding, liver and kidney problems, and medication costs. Additional considerations regarding choice of treatment noted in the literature include risk of common side effects such as nausea and heartburn, likelihood of benefit in terms of decreased pain and improved function, possible interactions with co-existing conditions or other medications, speed of onset of pain relief, and method of administration [34,35]. A patient focus group conducted at the beginning of the study identified several additional factors affecting choice of osteoarthritis pain medication, including specific concerns about possible cardiovascular side effects and medication administration issues such as the number of daily doses and when pain medications should be taken relative to food and other drugs. Based on this information, we included the following medication characteristics in the prototype decision dashboard: effectiveness in relieving pain, risk of side effects, possibility of drug-drug interactions, out-of-pocket cost, and how the drug is administered.

To allow for comparisons between the dashboard prototype and the AHRQ brochure we included the same treatment options in the dashboard. Data needed to describe the drug choices relative to each decision criterion were obtained as follows:

•We used the overall effect sizes from a meta-analysis published shortly before we created the dashboard prototype to summarize effectiveness [36].

•We estimated side effect risks using information obtained from the comparative effectiveness review that served as the basis for the AHRQ decision aid, a meta-analysis of osteoarthritis pain medications, and the MEDEX^®^ drug database [33,36,37]. We defined low risk as no serious side effects and three or fewer common side effects, moderate risk as one to three serious side effects or four to ten common side effects, and high risk as either four or more serious side effects or more than ten common side effects.

•We obtained information about possible drug-drug interactions from the Lexi-Comp Online™ Interaction Lookup database [38]. We defined low risk as no known interactions, moderate risk as interactions possible but not thought to be clinically important, and high risk as some interactions possible that could affect patient care.

•Cost estimates, recommended dosing schedules, and routes of administration were obtained from the Tarascon Pharmacopoeia, Mobile version [39]. We defined low cost as patient out-of-pocket expenses of $5 to $10 per month, moderate cost as $11 to $25 per month, high cost as $26 to $50 per month, and very high cost as monthly out-of-pocket expenses of more than $50.

Table 1 summarizes the treatment-related information included in the dashboard prototype. This information was current at the time of the study (2008–09). To avoid respondent bias due to past treatment experiences or name recognition, we identified the options on the dashboard using arbitrary letters rather than the actual drug names. Note that the data indicate that two options, - non-steroidal anti-inflammatory drugs plus misoprostol and non-steroidal anti-inflammatory drugs and proton pump inhibitors - can be considered inferior choices because other treatment options are available that are better with respect to every medication characteristic being considered.

**Table 1:** Drug-related information included in the dashboard

**Drug (Dashboard abbreviation)**	Pain relief *	**Risk of side effects**	**Risk of drug-drug interactions**	**Cost**	**Administration**
Acetaminophen (a)	0.21	Low	Moderate	Low	1 tablet every 6 hours
Topical NSAID (b)	0.41	Low	Low	High	Cream twice a day
Capsaicin (c)	0.30	Low	Low	Moderate	Cream twice a day
NSAID + misoprostol (d)	0.32	High	High	Moderate	2 tablets twice a day
NSAID + PPI (e)	0.32	Moderate	High	Very High	2 tablets twice a day
NSAIDs (f)	0.32	Moderate	High	Low	1 tablet twice a day
Celecoxib (g)	0.44	Moderate	High	High	1 tablet twice a day
Chondroitin sulfate (h)	0.30	Low	Low	Moderate	1 tablet twice a day
Glucosamine sulfate (i)	0.45	Low	Moderate	Moderate	1 tablet twice a day

* - Pain relief summarized using reported effect size.

Abbreviations: *NSAID* = non-steroidal anti-inflammatory drug; *PPI* = proton pump inhibitor.

**Figure 1:**
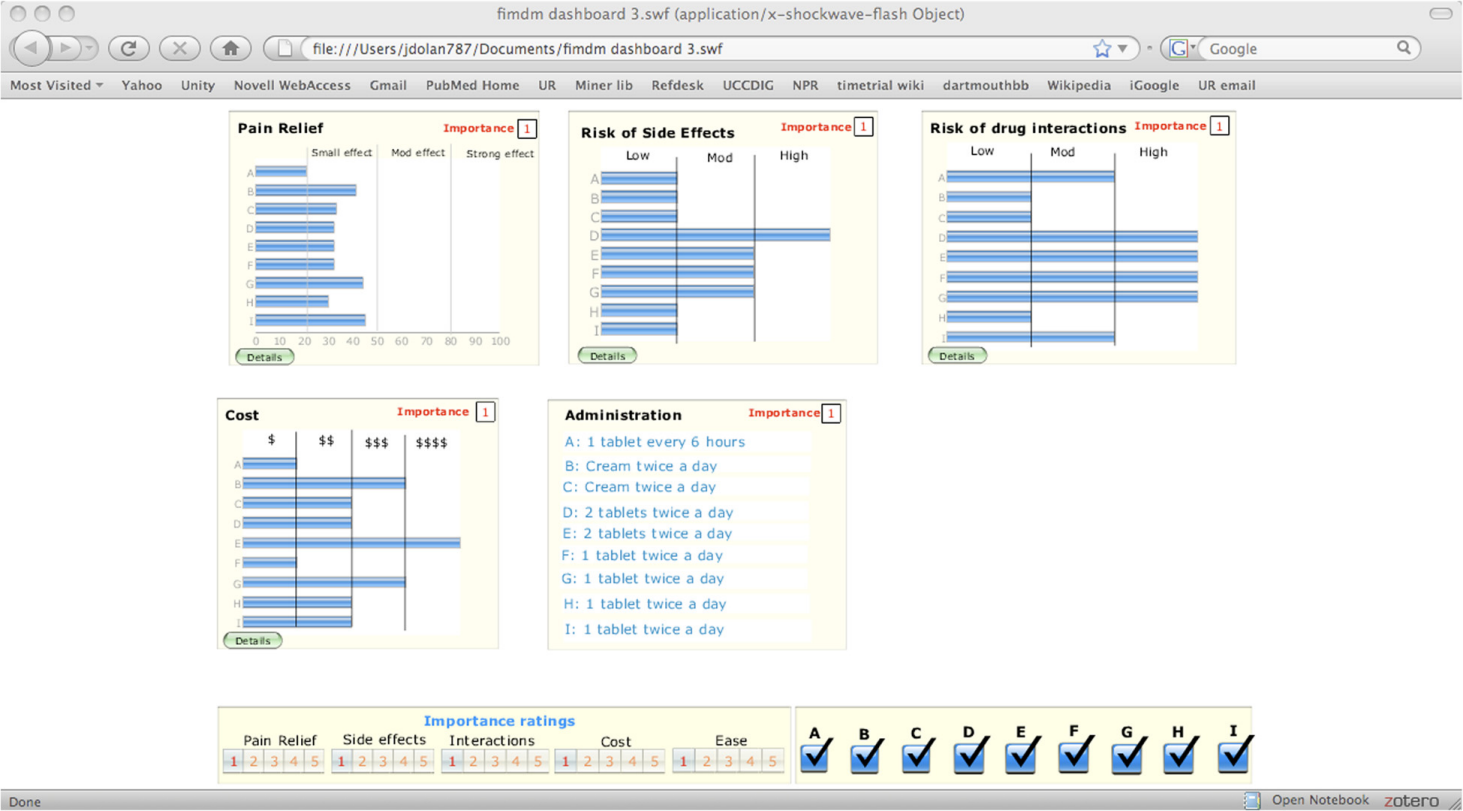
The decision dashboard.

The main screen of the resulting dashboard prototype is shown in Figure 1. It consists of five windows that summarize the relative performance of the treatment alternatives with regard to each of the included drug information categories: effectiveness in relieving pain, risk of side effects, possibility of drug-drug interactions, out-of-pocket cost, and how the drug is administered. Buttons for obtaining additional category-specific information are included within the window for every category except administration. There are also buttons at the bottom of the display that can be used to prioritize the importance of each drug information category in making a treatment choice and to determine which drugs are included in the display.

We designed the dashboard prototype following guidelines proposed by Few [13] and programmed it using *Microsoft Excel* and *Crystal Xcelsius*, a program designed to create interactive decision dashboards from *Excel* files [40,41].

### Prototype dashboard assessment

We tested the usability and effectiveness of the clinical dashboard prototype by recruiting study volunteers from nursing and secretarial staff working at Unity Faculty Partners (a general internal medicine resident/faculty teaching practice affiliated with Unity Health System in Rochester NY), patients from the same practice, Unity Hospital Department of Medicine support staff, and volunteers responding to a notice about the study posted on the University of Rochester Medical Center clinical trials website. The study was conducted over a three month period between February 12 and May 5, 2009.

After a brief introduction, study participants were asked to: a) imagine they had developed symptomatic osteoarthritis in their right knee that was interfering with their usual activities, b) that their physician had asked them to use the dashboard to review possible initial treatment options in preparation for an upcoming appointment and choose a preferred medication, and c) after a brief instructional demonstration, to use it to help them identify a preferred treatment option. All participants individually reviewed the dashboard running on a personal computer equipped with a touch screen monitor in a private setting.

If a participant actually had arthritis, we asked them to make their treatment choice as if they had no arthritic problems other than the knee pain that was described. Otherwise, we instructed study participants to select a preferred treatment option based on their actual personal circumstances and preferences.

### Evaluation

We evaluated the potential usefulness of the clinical dashboard format for creating clinically realistic and feasible patient decision aids using a multi-method procedure that included observations of how the study participants used the dashboard, measures designed to assess ease of use, acceptability, and decisional conflict, and an open-ended qualitative analysis.

The observational data included the time the participants spent using the dashboard before choosing a preferred drug, which drug they chose, and whether they used three optional features included in the dashboard: the touch screen option, the priority boxes assigned to each consideration, and the opportunity to remove information about specific drugs from the dashboard display.

The remainder of the evaluation was conducted after the participants had finished using the dashboard and selected their preferred drug. The first part consisted of a 21 item questionnaire designed to measure the dashboard’s ease of use and acceptability derived from two validated instruments to measure users’ evaluations of computer-based tools: the Unified Theory of Acceptance and Use of Technology (UTAUT) [42] and the WebQual instrument [43]. We used items from both scales chosen to eliminate redundancy and adapted them to fit a medical treatment decision context. The resulting scale included four component sub-scales: mechanical ease of use (4 items), cognitive ease of use (7 items), emotional difficulty (3 items), and decision-aiding effectiveness (7 items). Possible responses to all items consisted of a 7-point scale ranging from strongly disagree to strongly agree. Sub-scale scores were calculated using the mean item response. The resulting scale is shown in Table 2.

**Table 2:** Quantitative outcome measures *

**Scale component**	**Items**
**Ease of use, mechanical**	a. I found the program easy to use
	b. It was easy to find information and move through the program.
	c. The design of the program was appropriate.
	d. I think I could learn to use the program on my own.
**Ease of use, cognitive**	a. I found the program clear and easy to understand.
	b. The program provides accurate information.
	c. The program provides believable information.
	d. The program provides relevant information.
	e. The program provides easy to understand information.
	f. The program provides information at the right level of detail.
	g. The program provides information in an appropriate format.
**Ease of use, emotional**	a. I felt nervous using the program.
	b. I would not wish to use the program to help with my medical care because I am afraid I would make mistakes.
	c. The program was intimidating to me.
**Decision-aiding effectiveness**	a. I would find this program useful in treating my arthritis pain.
	b. Using this program would help me learn about my treatment options more quickly.
	c. Using this program would increase my chances of controlling my arthritis pain safely and effectively.
	d. If I could, I would use this program.
	e. I think the program would make it easier for me to talk to my doctor about my arthritis pain treatment.
	f. I feel confident that the program would help me treat my arthritis pain better.
	g. The program would help me get the arthritis treatment that is best for me.
**Decisional conflict scale, informed sub-scale**	a. I know what options are available to me for treating my arthritis pain.
	b. I know the benefits of each option.
	c. I know the risks & side effects of each option.
**Decisional conflict scale, values clarification sub-scale**	a. I am clear about which benefits matter most to me.
	b. I am clear about which risks and side effects matter most to me.
	c. I am clear about which benefits, risks, and side effects matter most to me.
**Decisional conflict scale, uncertainty sub-scale**	a. I am clear about the best choice for me.
	b. I feel sure about what to choose.
	c. The decision is easy for me to make.

* Possible responses to all items consisted of a 7-point scale ranging from strongly disagree to strongly agree. Sub-scale scores were calculated using the mean item response.

We assessed the effect of the dashboard on participants’ decision making processes using the informed, values clarity, and uncertainty sub-scales from the decisional conflict scale modified to use a 7 rather than a 5 point scale to be consistent with the other assessment questions [44].

The qualitative analysis consisted of a series of open-ended questions exploring aspects of the participants’ experiences using the dashboard for the task of picking their preferred arthritis pain medication and to compare the dashboard with the conventionally formatted AHRQ booklet. The questions included in this phase of the analysis are shown in Additional files 1 and 2.

We used participant self-reports to determine age, racial background, and highest educational level attained. We measured health-related literacy and numeracy using the Rapid Estimate of Adult Literacy in Medicine (REALM), the subjective numeracy scale, and the Newest Vital Sign. The REALM is a test of word recognition that correlates well with other commonly used health literacy measures. Results are reported as grade-level equivalents [45]. The subjective numeracy scale consists of eight items and does not require any calculations. Scores range from 1 to 6 with higher scores indicating greater numeracy skills [46]. The Newest Vital Sign involves reading and interpreting information provided about the nutritional information of a container of ice cream. It has been shown to be a quick and reliable assessment of health literacy in primary care settings [47].

The study was approved by the institutional review boards of the University of Rochester and Unity Health System.

### Data analysis

We summarized quantitative data using standard descriptive statistical methods and evaluated the qualitative data using a thematic approach. The reliability of the outcome questionnaires was assessed using Cronbach’s alpha. All statistical analyses were done using *MedCalc 12*[48].

## Results

### Study sample

The characteristics of the study sample are summarized in Table 3. The majority were white women with at least an Associate’s degree and good to excellent literacy and numeracy skills. They were recruited in almost equal proportions from office and departmental staff, patient volunteers, and clinical trial website respondents.

**Table 3:** The study sample

**Variable**	**Number (percent)**
Gender	Male: 7 (28%)
	Female: 18 (72%)
Racial/ethnic background	White: 19 (76%)
	African-American: 2 (8%)
	Hispanic: 1 (4%)
	Asian: 3 (12%)
Highest Education level	High School or less: 3 (12%)
	Some college, no degree: 3 (12%)
	Associate’s degree: 11 (44%)
	Bachelor’s degree: 4 (16%)
	Post-graduate training: 4 (16%)
Recruitment source	Office staff: 7 (28%)
	Website volunteer: 8 (32%)
	Practice volunteer: 6 (24%)
	Department staff volunteer: 4 (16%)
Newest Vital Sign Health Literacy category	Adequate literacy: 18 (72%)
	Possible limited literacy: 5 (20%)
	High likelihood limited literacy: 2 (8%)
REALM grade level	High School: 21 (84%)
	7th – 8th grade: 3 (12%)
	4th to 6th grade: 1 (4%)
	**Mean (sd, range)**
Age, years	51.4, (13.8, 22 to 71)
Subjective numeracy scale	4 (0.75, 2.25 to 5.38)

### Dashboard use

The average time participants spent interacting with the dashboard was 4.6 minutes (range 0.4 to 11.7). The most commonly used extra feature of the dashboard was the option to delete the display of information about individual treatment options which was utilized by 14 (56%) participants. The other extra features were used less often. Five (25%) participants used the importance boxes and two (10%) of twenty who had the opportunity to use a touchscreen used it instead of a mouse to interact with the dashboard. (A working touchscreen was not available for the other five participants).

### Decision outcomes

The most commonly preferred drug was chondroitin sulfate, selected by 11 (48%) participants. The drug preferences of the other 14 participants were divided across six of the remaining eight options. Overall, seven (78%) of the nine treatment alternatives included in the dashboard were preferred by at least one participant. Only one patient selected an apparently inferior treatment choice (non-steroidal anti-inflammatory drugs and proton pump inhibitor, NSAIDS + PPI).

### Quantitative decision making process evaluations

**Figure 2:**
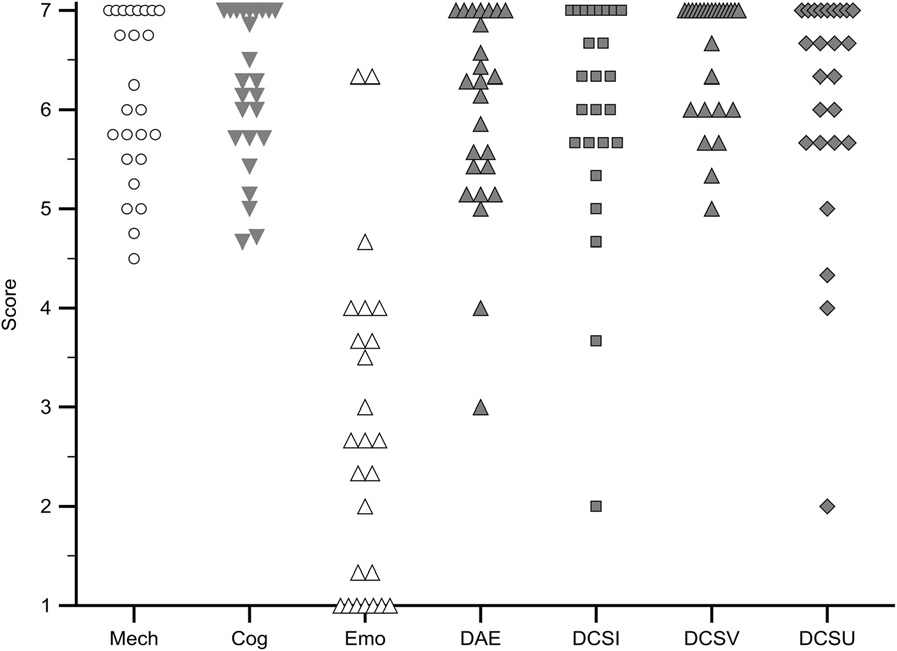
**Results of the quantitative decision making process assessment.** Dotplots showing the scores representing the mean quantitative assessment responses for the sub-scales used for the quantitative decision making process assessment. Abbreviations Mech = mechanical ease of use scale (4 items); Cog = cognitive ease of use scale (7 items); Emo = emotional ease of use scale (3 items); DAE = decision aiding effectiveness scale (7 items); DCSI = decisional conflict scale, informed sub-scale; DCSV = decisional conflict scale, values sub-scale; DCSU = decisional conflict scale, uncertainty sub-scale.

The results of the quantitative evaluation are summarized in Figure 2. Detailed results are included in a Additional files 1 and 2. The response rates were 100% for 17 (57%) of the 30 individual questions, 96% for 7 questions, and 92% for the other 6. The responses are consistently positive for questions concerning mechanical and cognitive ease of use, decision aiding effectiveness, and effectiveness in reducing decisional conflict by providing needed information, clarifying values, and easing uncertainty. There was no evidence of adverse emotional consequences. The Cronbach’s alpha coefficient for the 21-item adapted ease of use and acceptability scale was 0.90; for the three combined decisional conflict subscales it was 0.89.

### Qualitative decision making process evaluation

All participants answered the open-ended questions. By and large, they reported finding the computerized dashboard easy to use and helpful in evaluating medication choices for osteoarthritis pain and, potentially, for treatment decision-making more generally. Participants appreciated the simplicity of the tool, the graphic display of information, and the ability to compare specific treatment details and features via the side by side display (as well as the ability to “turn off” information they deemed personally irrelevant or unimportant). Some were surprised by the ways the dashboard revealed and helped prioritize their values regarding specific kinds of pain medication, their administration, and their cost.

Some respondents desired more evidence-based information about specific medications, including how long they had been in use, potential serious or long-term side effects, and how the drugs actually work in the body (including how quickly they take effect). Additionally, some questioned the source of the information (about the different medications) displayed on the dashboard and its accuracy.

Uniformly, respondents expressed that they would like ultimate choices about their pain medication to be made in collaboration with a doctor. In other words, they would not want to rely on their own judgment, even if assisted by a brochure or a computerized aid, to come to treatment decisions. Rather, they saw the utility of the computerized aid as educational and preparatory for their discussions with a doctor which would lead to final decisions about treatment choices through a shared decision making process.

When asked to choose between using the dashboard and the AHRQ brochure as a decision aid, 12 (48%) participants preferred the dashboard, 5 (20%) preferred the AHRQ brochure, and 8 (32%) preferred a combined approach.

## Discussion

These results suggest that the interactive decision dashboard format can be adapted to serve as a patient decision aid. The majority of our study participants were able to use the clinical dashboard prototype to work through a complicated decision problem in a remarkably efficient manner with excellent results in terms of ease-of-use, information provided, clarification of decision-related values, resolution of uncertainty about the treatment choice, and overall usefulness. As demonstrated by the positive responses to the usability and decisional conflict scales, we did not find evidence that the dashboard induced information overload. In fact, participants’ frequent use of the dashboard to eliminate less desirable options to focus attention on more promising alternatives suggests that the dashboard format provided a useful way for them to work through a large amount of information without being overwhelmed by it. There was no evidence of adverse emotional effects. A number of participants specifically commented that they found the dashboard display especially useful in identifying the key trade-offs involved in the decision, and 80% felt that a decision dashboard would be a valuable educational tool, either alone or in conjunction with complementary written material, to prepare them to participate in a shared decision making process with their health care provider.

These results are consistent with those of previous studies that have examined important features of the interactive dashboard format in isolation. Comparative studies have shown graphic formats more effective than numeric information for comparing the likelihoods of different outcomes, minimizing decision biases due to vivid anecdotal information, and promoting understanding of information by patients, including those over 75 years of age [49-53]. These studies all provide evidence supporting the theoretical advantages of visual information formats for supporting human decision making processes. There is also evidence that data about alternatives presented in a single side by side view promotes more effective comparisons and differentiation among decision alternatives [8,54-56]. Finally, positive results have been found using patient decision boards - which are essentially static, non-interactive dashboards - to support clinical decision making in Oncology [57,58].

This study has several limitations. Because the decision task involved a hypothetical situation, participant responses were not made in the context of making an actual clinical decision. The study sample was a small convenience sample of volunteers and the stability of their preferences was not assessed. Consequently, the results of this study may not adequately reflect findings from the general patient population. Finally, we made no attempt to assess how to integrate a clinical decision dashboard into routine patient care. None of these limitations, however, negates the primary finding of the study, i.e., that the interactive dashboard format can be successfully adapted to create a patient decision aid capable of quickly and efficiently helping at least some people identify preferred decision alternatives based on a large amount of complex data.

It is important to note that this study was designed to assess the feasibility of creating a patient decision aid using the interactive dashboard format rather than to evaluate the effectiveness of this approach or examine treatment preferences for osteoarthritis pain. Consequently, additional research is needed to determine effectiveness in a general patient population. Moreover, additional studies are needed to determine the amount and type of information clinical decision dashboards should contain, how to effectively involve busy practitioners in their use, and to explore the efficiency, effectiveness, and cost-effectiveness of using interactive dashboards to support patient-centered shared decision making in routine practice settings.

## Conclusions

In conclusion, these results suggest that an interactive clinical decision dashboard, either used alone or in conjunction with more traditional print-based reference materials, has the potential to be an effective and efficient format for creating clinical decision aids capable of fostering informed patient decision making and patient-centered care.

## Abbreviations

AHRQ: Agency for Heathcare Research and Quality; UTAUT: Unified Theory of Acceptance and Use of Technology; REALM: Rapid Estimation of Literacy in Medicine; NSAIDS: Non-Steroidal Anti-inflammatory Drugs; PPI: Proton pump inhibitors

## Competing interests

The authors declare they have no competing interests.

## Authors’ contributions

JD conceived the study, helped design it and the dashboard prototype, collected and analyzed the non-qualitative data, and drafted the manuscript. PV helped conceive and design the study and assisted with manuscript preparation. AR helped conceive and design the study, performed the qualitative analysis, and assisted with manuscript preparation. All authors read and approved the final manuscript.

## Pre-publication history

The pre-publication history for this paper can be accessed here:

http://www.biomedcentral.com/1472-6947/13/51/prepub

## Supplementary Material

Additional file 1 Qualitative evaluation questions. Click here for file

Additional file 2 Results of evaluation questionnaire. Click here for file
