# Supplementary material for: Development and initial evaluation of a treatment decision dashboard
Source: BMC Med Inform Decis Mak. 2013 Apr 21;13:51. doi: 10.1186/1472-6947-13-51 (PMC3639808; doi:10.1186/1472-6947-13-51)
Supplement: Additional file 1 — Qualitative evaluation questions. [file 1472-6947-13-51-S1.docx]

# Qualitative evaluation questions

| What could have made the program more directly relevant to you, and to your concerns in choosing a pain medication? |
| --- |
| Was there information that you wanted or needed that wasn't provided? |
| Did going through the aid bring up any questions for you? |
| What was especially helpful about the program? |
| Was anything particularly unhelpful? |
| Was there anything unsettling or upsetting |
| Did the sequence and flow of information make sense to you? |
| Was there anything that seemed unimportant? |
| Do you trust the aid? |
| Can you imagine using this aid (or the results) in conversation/collaboration with your doctor? |
| In the end, would you prefer your doctor make the decision about pain medication, or do you prefer to be in charge of that? |
| Please take a moment to look at this brochure. Like the computer program, it is designed to help patients learn more about treatments for arthritis pain. When you are done, please tell me which format you like better: the brochure or our computer program. What made you decide to favor one format over the other? |
