# Supplementary material for: Development and initial evaluation of a treatment decision dashboard
Source: BMC Med Inform Decis Mak. 2013 Apr 21;13:51. doi: 10.1186/1472-6947-13-51 (PMC3639808; doi:10.1186/1472-6947-13-51)
Supplement: Additional file 2 — Results of evaluation questionnaire. [file 1472-6947-13-51-S2.doc]

# Results of evaluation questionnaire

| | | **Ease of use, mechanical** | **mean** | **sd** | **median** | **min** | **max** | **range** | **number** | **source** |  | | --- | --- | --- | --- | --- | --- | --- | --- | --- | --- | | Q1 | I found the program easy to use | 6.2 | 1.14 | 7.0 | 3 | 7 | 4 | 25 | WebQual | | Q2 | It was easy to find information and move through the program. | 6.2 | 1.15 | 7.0 | 3 | 7 | 4 | 25 | WebQual | | Q3 | The design of the program was appropriate. | 6.3 | 0.95 | 7.0 | 4 | 7 | 3 | 25 | WebQual | | Q5 | I think I could learn use the program on my own. | 5.8 | 1.78 | 7.0 | 1 | 7 | 6 | 25 | utaut | |  | **Category summary** | **6.1** |  |  |  |  |  |  |  | |  |  |  |  |  |  |  |  |  |  | |  | **Ease of use, cognitive** | **mean** | **sd** | **median** | **min** | **max** | **range** | **number** | **source** | | Q4 | I found the program clear and easy to understand. | 6.1 | 1.14 | 6.8 | 3 | 7 | 4 | 24 | WebQual | | Q6 | The program provides accurate information | 6.2 | 1.15 | 7.0 | 4 | 7 | 3 | 25 | WebQual | | Q7 | The program provides believable information. | 5.8 | 1.35 | 6.0 | 3 | 7 | 4 | 24 | WebQual | | Q8 | The program provides relevant information. | 6.1 | 1.14 | 6.5 | 4 | 7 | 3 | 24 | WebQual | | Q9 | The program provides easy to understand information. | 6.3 | 1.00 | 7.0 | 3 | 7 | 4 | 24 | WebQual | | Q10 | The program provides information at the right level of detail. | 6.5 | 0.59 | 7.0 | 5 | 7 | 2 | 24 | WebQual | | Q11 | The program provides information in an appropriate format. | 6.4 | 0.72 | 6.5 | 5 | 7 | 2 | 24 | WebQual | |  | **Category summary** | **6.2** |  |  |  |  |  |  |  | |  |  |  |  |  |  |  |  |  |  | |  | **Ease of use, emotional** | **mean** | **sd** | **median** | **min** | **max** | **range** | **number** | **source** | | Q12 | I felt nervous using the program. | 3.1 | 2.48 | 1.5 | 1 | 7 | 6 | 24 | utaut | | Q13 | I would not want to use the program to help with my medical care because I am afraid I would make mistakes. | 2.6 | 1.73 | 2.0 | 1 | 6 | 5 | 25 | utaut | | Q14 | The program was intimidating to me. | 2.4 | 1.89 | 2.0 | 1 | 7 | 6 | 25 | utaut | |  | **Category summary** | **2.7** |  |  |  |  |  |  |  | |  |  |  |  |  |  |  |  |  |  | |  | **Decision-aiding effectiveness** | **mean** | **sd** | **median** | **min** | **max** | **range** | **number** | **source** | | Q15 | I would find this program useful in treating my arthritis pain. | 5.9 | 1.72 | 7.0 | 1 | 7 | 6 | 25 | utaut | | Q16 | Using this program would help me learn about my treatment options more quickly. | 5.8 | 1.26 | 6.0 | 3 | 7 | 4 | 25 | utaut | | Q17 | Using this program would increase my chances of controlling my arthritis pain safely and effectively. | 5.8 | 1.58 | 6.0 | 1 | 7 | 6 | 25 | utaut | | Q18 | If I could, I would use this program. | 6.2 | 0.98 | 7.0 | 4 | 7 | 3 | 23 | utaut | | Q19 | I think the program would make it easier for me to talk to my doctor about my arthritis pain treatment. | 6.2 | 1.92 | 7.0 | 4 | 7 | 3 | 23 | WebQual | | Q20 | I feel confident that the program would help me treat my arthritis pain better. | 5.8 | 1.23 | 6.0 | 2 | 7 | 5 | 23 | WebQual | | Q21 | The program would help me get the arthritis treatment that is best for me. | 5.9 | 1.35 | 6.0 | 2 | 7 | 5 | 23 | WebQual | |  | **Category summary** | **5.9** |  |  |  |  |  |  |  | |  | **Decisional conflict, informed sub-scale** | **mean** | **sd** | **median** | **min** | **max** | **range** | **number** | **source** | | Q22 | I know what options are available to me for treating my arthritis pain. | 6.1 | 1.25 | 7.0 | 2 | 7 | 5 | 23 | dcs | | Q23 | I know the benefits of each option. | 5.8 | 1.54 | 6.0 | 2 | 7 | 5 | 23 | dcs | | Q24 | I know the risks & side effects of each option. | 6.0 | 1.34 | 6.0 | 2 | 7 | 5 | 25 | dcs | |  | **Category summary** | **6.0** |  |  |  |  |  |  |  | |  |  |  |  |  |  |  |  |  |  | |  | **Decisional conflict, values clarification sub-scale** | **mean** | **sd** | **median** | **min** | **max** | **range** | **number** | **source** | | Q25 | I am clear about which benefits matter most to me. | 6.5 | 0.77 | 7.0 | 5 | 7 | 2 | 25 | dcs | | Q26 | I am clear about which risks and side effects matter most to me. | 6.6 | 0.65 | 7.0 | 5 | 7 | 2 | 25 | dcs | | Q27 | I am clear about which benefits, risks, and side effects matter most to me. | 6.6 | 0.58 | 7.0 | 5 | 7 | 2 | 25 | dcs | |  | **Category summary** | **6.5** |  |  |  |  |  |  |  | |  |  |  |  |  |  |  |  |  |  | |  | **Decisional conflict, uncertainty sub-scale** | **mean** | **sd** | **median** | **min** | **max** | **range** | **number** | **source** | | Q28 | I am clear about the best choice for me. | 6.3 | 1.37 | 7.0 | 1 | 7 | 6 | 25 | dcs | | Q29 | I feel sure about what to choose. | 6.1 | 1.30 | 6.0 | 1 | 7 | 6 | 25 | dcs | | Q30 | The decision is easy for me to make. | 5.9 | 1.33 | 6.0 | 3 | 7 | 4 | 25 | dcs | |  | **Category summary** | **6.1** |  |  |  |  |  |  |  | |  |  |  |  |  |  |  |  |  |  | | --- | --- | --- | --- | --- | --- | --- | --- | --- | --- | --- | --- | --- | --- | --- | --- | --- | --- | --- | --- | --- | --- | --- | --- | --- | --- | --- | --- | --- | --- | --- | --- | --- | --- | --- | --- | --- | --- | --- | --- | --- | --- | --- | --- | --- | --- | --- | --- | --- | --- | --- | --- | --- | --- | --- | --- | --- | --- | --- | --- | --- | --- | --- | --- | --- | --- | --- | --- | --- | --- | --- | --- | --- | --- | --- | --- | --- | --- | --- | --- | --- | --- | --- | --- | --- | --- | --- | --- | --- | --- | --- | --- | --- | --- | --- | --- | --- | --- | --- | --- | --- | --- | --- | --- | --- | --- | --- | --- | --- | --- | --- | --- | --- | --- | --- | --- | --- | --- | --- | --- | --- | --- | --- | --- | --- | --- | --- | --- | --- | --- | --- | --- | --- | --- | --- | --- | --- | --- | --- | --- | --- | --- | --- | --- | --- | --- | --- | --- | --- | --- | --- | --- | --- | --- | --- | --- | --- | --- | --- | --- | --- | --- | --- | --- | --- | --- | --- | --- | --- | --- | --- | --- | --- | --- | --- | --- | --- | --- | --- | --- | --- | --- | --- | --- | --- | --- | --- | --- | --- | --- | --- | --- | --- | --- | --- | --- | --- | --- | --- | --- | --- | --- | --- | --- | --- | --- | --- | --- | --- | --- | --- | --- | --- | --- | --- | --- | --- | --- | --- | --- | --- | --- | --- | --- | --- | --- | --- | --- | --- | --- | --- | --- | --- | --- | --- | --- | --- | --- | --- | --- | --- | --- | --- | --- | --- | --- | --- | --- | --- | --- | --- | --- | --- | --- | --- | --- | --- | --- | --- | --- | --- | --- | --- | --- | --- | --- | --- | --- | --- | --- | --- | --- | --- | --- | --- | --- | --- | --- | --- | --- | --- | --- | --- | --- | --- | --- | --- | --- | --- | --- | --- | --- | --- | --- | --- | --- | --- | --- | --- | --- | --- | --- | --- | --- | --- | --- | --- | --- | --- | --- | --- | --- | --- | --- | --- | --- | --- | --- | --- | --- | --- | --- | --- | --- | --- | --- | --- | --- | --- | --- | --- | --- | --- | --- | --- | --- | --- | --- | --- | --- | --- | --- | --- | --- | --- | --- | --- | --- | --- | --- | --- | --- | --- | --- | --- | --- | --- | --- | --- | --- | --- | --- | --- | --- | --- | --- | --- | --- | --- | --- | --- | --- | --- | --- | --- | --- | --- | --- | --- | --- | --- | --- | --- | --- | --- | --- | --- | --- | --- | --- | --- | --- | --- | --- | --- | --- | --- | --- | --- | --- | --- | --- | --- | --- | --- | --- | --- | --- | --- | --- | --- | --- | --- | --- | --- | --- | --- | --- | --- | --- | --- | --- | --- | --- | --- | --- | --- | --- | --- | --- | --- | --- | --- | --- | --- | --- | --- | --- | --- | --- | --- | --- | --- | --- | --- | --- | --- | --- | --- | --- | --- | --- | --- | --- | --- | --- | --- | --- | --- | --- | --- | --- | --- | --- | --- | --- | --- | --- | --- | --- | --- | --- | --- | --- | --- | --- | --- | --- | --- | --- | --- | --- | --- | --- | --- | --- | --- | --- | --- | --- | --- | --- | --- | --- | --- | --- | --- | --- | --- | --- | --- | | Abbreviations: sd = standard deviation, utaut = Unified theory of acceptance and use of technology, dcs = decisional conflict scale. |  |  |  |  |  |  |  |  |  |  | |  |  |  |  |  |  |  |  |  |  |
| --- | --- | --- | --- | --- | --- | --- | --- | --- | --- | --- | --- | --- | --- | --- | --- | --- | --- | --- | --- | --- | --- | --- | --- | --- | --- | --- | --- | --- | --- | --- | --- | --- | --- | --- | --- | --- | --- | --- | --- | --- | --- | --- | --- | --- | --- | --- | --- | --- | --- | --- | --- | --- | --- | --- | --- | --- | --- | --- | --- | --- | --- | --- | --- | --- | --- | --- | --- | --- | --- | --- | --- | --- | --- | --- | --- | --- | --- | --- | --- | --- | --- | --- | --- | --- | --- | --- | --- | --- | --- | --- | --- | --- | --- | --- | --- | --- | --- | --- | --- | --- | --- | --- | --- | --- | --- | --- | --- | --- | --- | --- | --- | --- | --- | --- | --- | --- | --- | --- | --- | --- | --- | --- | --- | --- | --- | --- | --- | --- | --- | --- | --- | --- | --- | --- | --- | --- | --- | --- | --- | --- | --- | --- | --- | --- | --- | --- | --- | --- | --- | --- | --- | --- | --- | --- | --- | --- | --- | --- | --- | --- | --- | --- | --- | --- | --- | --- | --- | --- | --- | --- | --- | --- | --- | --- | --- | --- | --- | --- | --- | --- | --- | --- | --- | --- | --- | --- | --- | --- | --- | --- | --- | --- | --- | --- | --- | --- | --- | --- | --- | --- | --- | --- | --- | --- | --- | --- | --- | --- | --- | --- | --- | --- | --- | --- | --- | --- | --- | --- | --- | --- | --- | --- | --- | --- | --- | --- | --- | --- | --- | --- | --- | --- | --- | --- | --- | --- | --- | --- | --- | --- | --- | --- | --- | --- | --- | --- | --- | --- | --- | --- | --- | --- | --- | --- | --- | --- | --- | --- | --- | --- | --- | --- | --- | --- | --- | --- | --- | --- | --- | --- | --- | --- | --- | --- | --- | --- | --- | --- | --- | --- | --- | --- | --- | --- | --- | --- | --- | --- | --- | --- | --- | --- | --- | --- | --- | --- | --- | --- | --- | --- | --- | --- | --- | --- | --- | --- | --- | --- | --- | --- | --- | --- | --- | --- | --- | --- | --- | --- | --- | --- | --- | --- | --- | --- | --- | --- | --- | --- | --- | --- | --- | --- | --- | --- | --- | --- | --- | --- | --- | --- | --- | --- | --- | --- | --- | --- | --- | --- | --- | --- | --- | --- | --- | --- | --- | --- | --- | --- | --- | --- | --- | --- | --- | --- | --- | --- | --- | --- | --- | --- | --- | --- | --- | --- | --- | --- | --- | --- | --- | --- | --- | --- | --- | --- | --- | --- | --- | --- | --- | --- | --- | --- | --- | --- | --- | --- | --- | --- | --- | --- | --- | --- | --- | --- | --- | --- | --- | --- | --- | --- | --- | --- | --- | --- | --- | --- | --- | --- | --- | --- | --- | --- | --- | --- | --- | --- | --- | --- | --- | --- | --- | --- | --- | --- | --- | --- | --- | --- | --- | --- | --- | --- | --- | --- | --- | --- | --- | --- | --- | --- | --- | --- | --- | --- | --- | --- | --- | --- | --- | --- | --- | --- | --- | --- | --- | --- | --- | --- | --- | --- | --- | --- | --- | --- | --- | --- | --- | --- | --- | --- | --- | --- | --- | --- | --- | --- | --- | --- | --- | --- | --- | --- | --- | --- | --- | --- | --- | --- | --- | --- | --- | --- | --- | --- | --- | --- | --- | --- | --- | --- | --- | --- | --- | --- | --- | --- | --- | --- | --- | --- | --- | --- |
